# Comparing Simvastatin Monotherapy V/S Simvastatin-Ezetimibe Combination Therapy for the Treatment of Hyperlipidemia: A Meta-Analysis and Review

**DOI:** 10.7759/cureus.31007

**Published:** 2022-11-02

**Authors:** Dhruva Chauhan, Farzana Memon, Vaibhav Patwardhan, Priya Kotwani, Parth Shah, Vikramaditya Samala Venkata

**Affiliations:** 1 General Internal Medicine, Gujarat Cancer Society Medical College, Ahmedabad, IND; 2 Epidemiology and Public Health, Indian Institute of Public Health Gandhinagar, Ahmedabad, IND; 3 Public Health, Parul University, Vadodara, IND; 4 Monitoring, Learning and Evaluation, Jhpiego, New Delhi, IND; 5 Hospital Medicine, Tower Health Medical Group, West Reading, USA; 6 Department of Internal Medicine, Cheshire Medical Center, Dartmouth-Hitchcock, Keene, USA

**Keywords:** cardiovascular health, cholesterol, prevention in primary care, non-statin therapy, hyperlipidemia

## Abstract

Longstanding hyperlipidemia can increase the risk of cardiovascular disease. Statins are currently the mainstay of treatment in hyperlipidemia. Combination therapy of statin with ezetimibe is only indicated for severe hypercholesterolemia and very high-risk atherosclerotic cardiovascular disease (ASCVD) population. There is a paucity of studies comparing statin monotherapy vs combination therapy with ezetimibe. This study aims to perform a meta-analysis of the existing literature and compare the effectiveness of statin monotherapy with statin-ezetimibe combination therapy in the management of hyperlipidemia.

A systematic electronic search of the scientific literature was performed in PubMed, EMBASE, and Scopus. Only randomized controlled trials comparing simvastatin monotherapy vs simvastatin-ezetimibe combination therapy between the years 2000 and 2021 and published in English language were included. Fifteen studies were included in the final analysis. The main outcomes that were compared were a reduction in low-density lipoprotein (LDL) and high-density lipoprotein (HDL).

Our study showed that combination therapy led to a higher reduction of LDL-C (Mean difference: -20.22(-26.38, -14.07); P<0.0001) compared to monotherapy with a statin alone. There was no significant difference in the reduction of HDL-C values (Mean difference: -0.07(-0.45,0.32); P-0.04) between the two groups.

Our study indicates that the combination therapy of simvastatin and ezetimibe is more effective in reduction of LDL-C levels compared to simvastatin monotherapy alone. Currently, guidelines recommend combination therapy only for severe hypercholesterolemia and high-risk ASCVD patients, more studies are needed to study the effectiveness of simvastatin-ezetimibe combination therapy in low-risk ASCVD population.

## Introduction and background

Cardiovascular disease is the leading cause of death in the United States and around the world [1]. Hyperlipidemia can lead to nearly a fourfold increase in the risk of cardiac disease [2-3]. Hyperlipidemia is one of the most prevalent chronic medical conditions in the United States. According to a heart and stroke statistics-2020 update from the American Heart Association (AHA), 38% of the adult population had elevated TG levels (>200 mg/dl) and 29% had elevated low-density lipoprotein (LDL) levels(>130 mg/dl), causing a significant burden on the healthcare system and leading to increased mortality [3].

Statins are the mainstay of treatment in hyperlipidemia [4-5]. Statins work by inhibiting the enzyme hydroxymethylglutaryl-coenzyme-A reductase (HMGCR) which is used in the synthesis of cholesterol [6].

Ezetimibe, fibrates, niacin, and bile acid-binding resins are the other classes of drugs that can be used to treat hyperlipidemia [7]. Ezetimibe works by reducing cholesterol absorption in the small bowel [8]. As per current guidelines, combination therapy with statin and ezetimibe is recommended only for severe hypercholesterolemia and very high-risk atherosclerotic cardiovascular disease (ASCVD) populations [9-10]. There is currently a paucity of studies looking at the combination therapy of statin and ezetimibe compared to statin therapy alone. In this paper, we aim to perform a meta-analysis of existing literature and compare combination therapy of statin + ezetimibe to statin therapy alone.

## Review

Materials and methods

The Cochrane Handbook for Systematic Reviews of Interventions was used to plan and conduct this meta-analysis [11]. Results were reported as per the preferred reporting items for systematic reviews and meta-analyses (PRISMA) guidelines [12].

Search strategies

**Figure 1:**
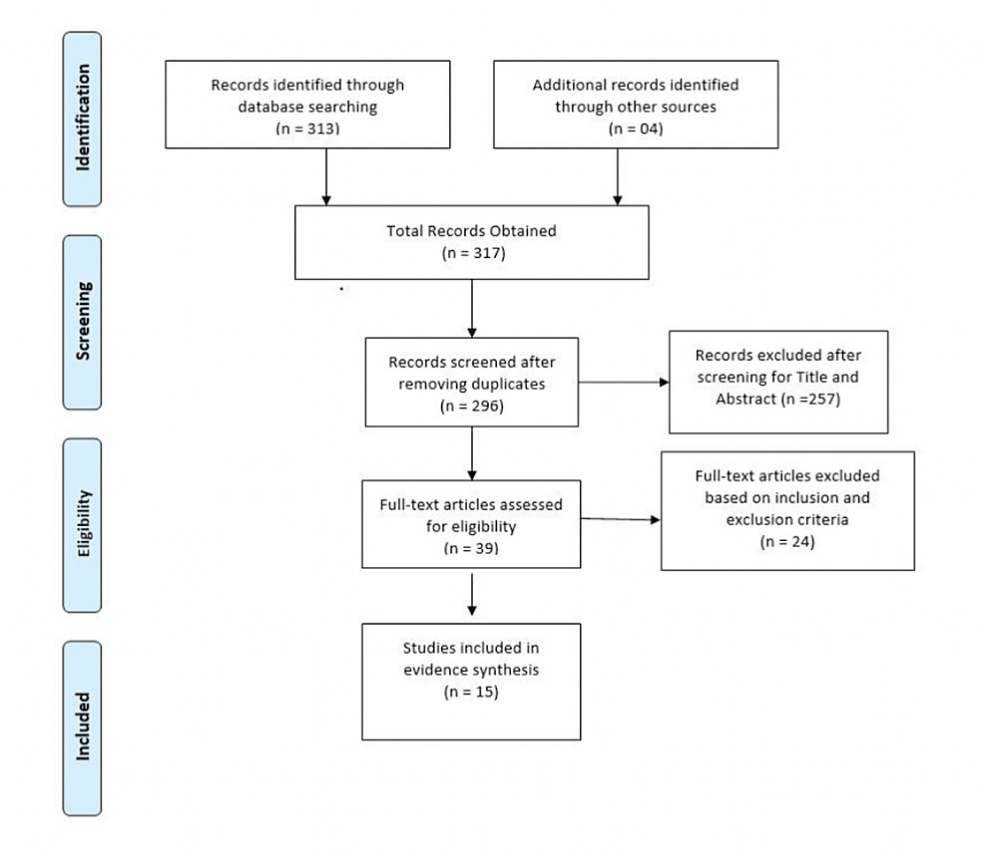
PRISMA diagram of study selection PRISMA: preferred reporting items for systematic reviews and meta-analyses

The literature search was performed using electronic databases: EMBASE, PubMed, and Scopus from 2000 to January 2021. The search terms used were: “Simvastatin” AND “Ezetimibe” AND “LDL-C” OR “low density lipoprotein” OR “hypercholesterolemia” OR “HDL” OR “High Density Lipoprotein” OR “Triglyceride” OR “TG” OR “Lipid Profile”. Only randomized controlled trials studying the changes of simvastatin versus simvastatin-ezetimibe combination drug on LDL, high-density lipoprotein (HDL), and triglycerides (TG) with outcomes reported in the form of mean values were included in the study. Only articles published in the English language between the years 2000 and 2021 were included. Additionally, the reference articles from recovered articles were checked to look for further significant studies. Initially, 317 citations were identified, of which 39 articles were reviewed for full text and finally 15 studies meeting inclusion and exclusion criteria were selected for inclusion in the meta-analysis [13-27]. Details of the process for selecting studies are given in Figure 1.

Data extraction 

Two authors DC and FM performed the final selection of studies for inclusion in the review independently. Differences of opinion were resolved by discussion among authors PK and VP.

The following methodological domains were considered: random sequence generation, allocation concealment, blinding of participants and personnel, blinding of outcome assessment, incomplete outcome data, selective reporting, and other potential threats to validity. We explicitly judged each of the domains as having high risk, low risk of bias, or studies showing some concerns. The data on the mean reduction of LDL, improvement in HDL, and reduction in TG for patients on simvastatin (control arm) and patients on combination therapy of simvastatin-ezetimibe drug (intervention arm) were extracted for all studies. Forest plots were used to display the mean change and its 95% CI for each study.

Statistical analysis

Heterogeneity calculations were performed with I2 indicating the level of heterogeneity (high: 75-100%, medium: 50-70%, and low: 0-50%). A two-tailed value of p<0.05 was considered as statistically significant where a fixed-effect model was used in cases where was below 50% and in cases where is above 50%, a randomized-effect model was adopted. Pooling of the data, where possible, was performed using Review Manager (RevMan) Version 5.4.1, Copenhagen: The Nordic Cochrane Centre, The Cochrane Collaboration, 2014. Graphical presentation of mean reduction in LDL and TG values, and mean improvement in HDL value are presented using forest plots. Publication bias was assessed using Begg’s test.

Results

**Figure 2:**
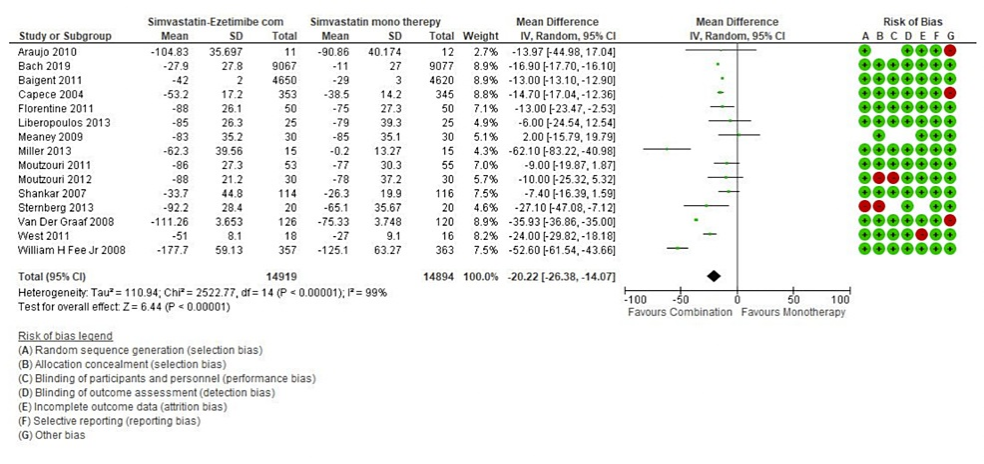
LDL forest plot LDL: low-density lipoprotein

**Figure 3:**
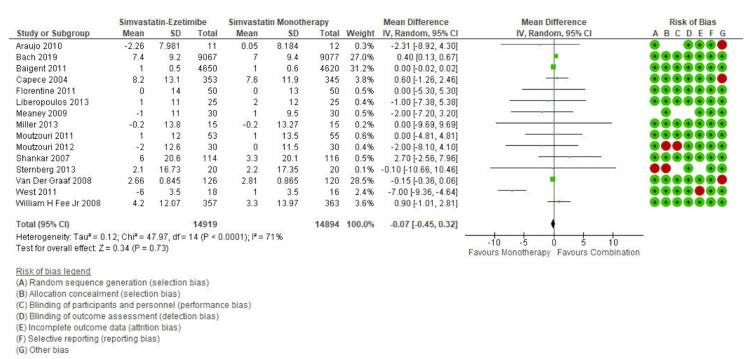
HDL forest plot HDL: high-density lipoprotein

The initial search revealed 317 articles of which 39 were reviewed in full, with 15 meeting inclusion criteria and were meta-analyzed (Figure 1). The overall pooled effect from 15 studies reporting the effect of simvastatin and simvastatin-ezetimibe combination showed that mean reduction in LDL values from the baseline values was higher in the combination therapy group compared to that in patients treated with simvastatin alone (Mean difference: -20.22 CI: -26.38 to -14.04; P<0.0001, I2: 99%) (Figure 2). However, when the effect of these drugs was studied on the improvement in the HDL values, it was observed that both simvastatin alone as well as simvastatin-ezetimibe combination drugs had almost the same effect on HDL values (Mean difference: -0.07 CI: -0.45 to 0.32; P=0.73; I2: 71%) (Figure 3).

Publication bias

The Begg’s test indicates publication bias for studies included in assessing the pooled effect of drugs in the control and intervention arm on LDL values. However, no publication bias was observed for studies reporting HDL and TG values. The results are highlighted in Table 1.

**Table 1:** Publication bias assessment using Begg’s test LDL: low-density lipoprotein; HDL: high-density lipoprotein; TG: triglycerides

Begg’s test (LDL)	
Kendall’s Tau	0.4286
Significance level	P = 0.0260
Begg’s test (HDL)	
Kendall’s Tau	-0.2952
Significance level	P = 0.1250
Begg’s test (TG)	
Kendall’s Tau	0.1111
Significance level	P = 0.6767

Discussion

Statins are currently the mainstay in the treatment of hyperlipidemia. With advances in medical research, novel agents like ezetimibe, colesevelam, torcetrapib, avasimibe, and implitapide have been considered in the treatment of hyperlipidemia [7]. As per current guidelines, ezetimibe, bile acid sequestrants, and PCSK9 inhibitors are the only non-statin agents that are recommended for the reduction of LDL levels. Ezetimibe works by reducing cholesterol absorption in the small bowel. The addition of ezetimibe to a statin regimen increases the magnitude of LDL-C lowering by approximately 13% to 20% [28]. With bile acid sequestrant agents and PCSK9 inhibitors, there are concerns regarding adverse events and an unknown long-term safety profile [29-30].

The evidence is not clear regarding the effectiveness of combination therapy of statin/ezetimibe over statin monotherapy alone. The ideal therapy must aim to achieve the LDL cholesterol goals established by the National Cholesterol Education Programme Adult Treatment Panel III (NCEP ATP III) [31]. While multiple recent studies demonstrated a significant reduction in LDL levels and attainment of LDL cholesterol goals with combination therapy when compared to monotherapy with statins alone [23,32-33] others suggested an equal effect of two therapies in lowering the LDL levels [13,18,19,34]. A study by Catapano et al. showing a benefit of combination therapy over statin monotherapy was a double-blinded multicenter study on randomized hypercholesteremic patients [32]. Our meta-analysis results suggested a pooled reduction in LDL-C levels by an additional 20.22 mg/dl (95% CI: 26.38 to 14.02; p< 0.00001) using combination therapy when compared with monotherapy (Figure 2).

Gagné et al. in their study found that ezetimibe when given in combination with statins yield positive results with a more substantial change in HDL-C (+2.7%) levels when compared to the monotherapy group (HDL-C +1%) along with significant reduction of LDL levels [35]. Similar findings were reported by Davidson et al. [36] and Gaudiani et al. [37] in their multicenter RCT conducted in type 2 diabetic patients. Our study findings suggest no significant difference in improvement of HDL level (Mean difference: -0.07; CI: -0.45 to 0.32; P=0.73) across both groups (Figure 3).

Moreover, the safety and tolerance of ezetimibe-simvastatin combination therapy were found to be equally safe as that of monotherapy across both genders and specific age groups [16,38,39].

Limitations

The articles found in our search reported different units of measurement to represent LDL-C, TG, and HDL-C values. One of the limitations of our meta-analysis is the inclusion of only those articles which reported the LDL-C, TG, and HDL-C levels in mg/dl. Another limitation would be that the included studies did not have a uniform dose for both therapy groups and used varying doses for their trials. Additionally, the effect of statin versus simvastatin-ezetimibe combination therapy on very-low-density lipoprotein (VLDL) was not studied in our analysis.

## Conclusions

Our study indicates that the dual inhibitory effect provided by simvastatin-ezetimibe combination therapy might be more effective in lowering LDL levels when compared to simvastatin monotherapy. There was no significant difference in HDL-C levels between both groups. The combination therapy of simvastatin-ezetimibe has the potential to be considered an effective and safe treatment modality for the management of hypercholesterolemia. More randomized controlled multicenter trials are needed to assess the effectiveness of combination therapy with statin and ezetimibe when compared to statin monotherapy alone.
